# Fetuin-A expression in human umbilical vein endothelial cells and amnion cells of patients with gestational diabetes mellitus

**DOI:** 10.15537/smj.2022.43.7.20220283

**Published:** 2022-07

**Authors:** Selim Afsar, Ayse Yigit, Ruhsen Ozcaglayan, Ceyda S. Usta, Cagla B. Bulbul, Gulay Turan

**Affiliations:** *From the Department of Obstetrics and Gynecology (Afsar, Usta, Bulbul); from the Department of Internal Medicine (Ozcaglayan); from the Department of Pathology (Turan), School of Medicine, Balikesir University, Balikesir, and from the Department of Obstetrics and Gynecology (Yigit), Adana Yuregir Devlet Hastanesi, Adana, Turkey.*

**Keywords:** gestational diabetes, fetuin-A, human umbilical vein endothelial cell, amniotic membrane

## Abstract

**Objectives:**

To elucidate the link between fetuin-A expression in human umbilical vein endothelial cells (HUVECs) and amnion cells (ACs) and clinicopathological changes in patients with gestational diabetes mellitus (GDM) and newborns.

**Methods:**

This retrospective cohort study included 82 pregnant patients (40 with GDM and 42 controls) between January 2019 and January 2022. The patients underwent a one-hour, 50 gram glucose challenge test (GCT) during the 24-28th weeks of pregnancy. Patients with positive GCTs immediately underwent a 3-hour, 100 gram oral glucose tolerance test. The expression level of fetuin-A in UVECs and ACs was evaluated by immunohistochemistry (IHC) and scored based on IHC staining in randomly selected slides. The IHC staining intensity was evaluated by the number of dots, which reﬂects the expression level of fetuin-A in both HUVECs and ACs.

**Results:**

The GDM group displayed significantly higher fetuin-A expression in both HUVECs (*p*<0.0001) and ACs (*p*=0.0001) when compared with the control group. Fetuin-A expression in HUVECs was correlated with fetal macrosomia, neonatal hypoglycemia, and placental weight. However, there was no correlation with fetuin-A expression in ACs.

**Conclusion:**

There is a correlation between fetal macrosomia, neonatal hypoglycemia, placental weight, and fetuin-A expression of HUVECs in patients with GDM.

**G**estational diabetes mellitus (GDM) is becoming more frequent and its prevalence is increasing in parallel with the obesity epidemic and coronavirus diseas-19 (COVID-19) pandemic.^1,2^ Gestational diabetes mellitus is seen with a prevalence of 5-10% of all pregnancies.^1^ In normal pregnancy, pancreatic B-cell hyperplasia occurs that results in higher insulin levels. Growth hormone, corticotropin releasing hormone, human placental lactogen, and progesterone are diabetogenic hormones secreted from placenta and all are related with insulin resistance. The inability to overcome this insulin resistance despite B-cell hyperplasia leads to GDM. Gestational diabetes mellitus is related with maternal and neonatal unfavorable consequences.^3^

Fetuin-A (α2 - Heremans-Schmid glycoprotein) which was first isolated from fetal bovine serum, functions as an important component of diverse normal and pathological processes, including vascular calcification, insulin resistance, bone metabolism regulation, protease activity control, neurodegenerative pathways, and tumor cell proliferative signaling.^4^

Fetuin-A is an inhibitor of insulin receptor tyrosine kinase and also it is associated with insulin resistance, type 2 diabetes and metabolic syndrome.^5,6^ In vitro and in vivo studies showed that fetuin-A inhibits insulin signaling and induces adipocyte dysfunction and it has distinct roles in the pathophysiology of insulin resistance, obesity, and nonalcoholic fatty liver disease.^7^ Fetuin-A seems to act as a regulatory glycoprotein for toll-like receptor 4 and it might provoke the proinflammatory pathways and insulin resistance in adipose tissue that eventually leads to type 2 diabetes.^8^ On the contrary in response to acute inflammation fetuin-A plays as an anti-inflammatory molecule.^9^

Fetuin-A and GDM are closely related entities. Gestational diabetes mellitus was not found to be associated with cord blood fetuin-A levels but fetuin-A was negatively associated with fetal growth in GDM. It was thought that fetuin-A could be a biomarker to predict the risk of GDM.^10^ In some papers, it was speculated that the levels of fetuin-A is higher in women with GDM. However, the function of fetuin-A in the placenta is nebilous.^11^ Fetuin might supress trophoblastic growth and ciliogenesis in patients with GDM.^12^ Moreover, the mothers of macrosomic infants had lower fetuin-A levels than the mothers of normal weighted babies.^13^

In literature, there are myriad papers which explores the correlation of serum fetuin-A levels and GDM, however; data is limited on the tissue expression of this molecule, especially on placental subunits of patients with GDM.^14,15^ Our purpose was to delineate the correlation between fetuin-A expressions in human umbilical vein endothelial cells (HUVECs) and amnion cells (ACs) in patients with GDM and clinicopathological variables of those patients and neonates.

## Methods

This retrospective cohort study was carried out between January 2019 and January 2022. The design of the study in parallel with the requirements of the Helsinki Committee essentials and it was approved by the Local Ethical Committee of the Balikesir University, School of Medicine, Balikesir, Turkey, and the informed consent was obtained from all participants.

Women who had singleton pregnancy underwent the 2-step approach to detect GDM and they were followed until delivery. Fasting blood glucose levels were measured after an 8-10 hours overnight fasting. They were screened with a one-hour, 50 grams glucose challenge test (GCT) during the 24^th^-28^th^ weeks of pregnancy. Those with (+) GCT result (glucose ≥140 mg/dl) proceeded to a diagnostic 3-hour, 100-grams oral glucose tolerance test (OGTT). Women with (-) GCT result were included in the control group. Those with ≥2 elevated values on a 3-hour, 100 gram OGTT (according to Carpenter and Coustan criteria) were included in GDM group.^1^

Based on GCT or OGTT results 82 age-matched patients were included into the study as GDM group (n=40) or control group (n=42) and those placental tissue and amnion membranes were retrieved after delivery. Patients with pregestational diabetes, multiple pregnancies, history of hypertension, other chronic systemic diseases, intrauterine infections, and fetal anomaly were excluded from the study.

After placental specimens were assessed morphologically for placental weight and cord insertion site, those were fixed and embedded in paraffin for detailed histopathological examination. Four micron sections of HUVECs and amniotic membranes were dried and slides were exposed to phosphate buffered saline (PBS). Each slide was rinsed with 0.1% Triton-X-100 and were buffered with citrate solution to ease antigen uptake. Afterwards, anti-fetuin-A antibodies (Santa Cruz Biotechnology Inc, Dallas Tx, USA) were introduced to the slides and tissue extracts were then rinsed again with PBS and were stained with hematoxyline.

**Figure 1:**
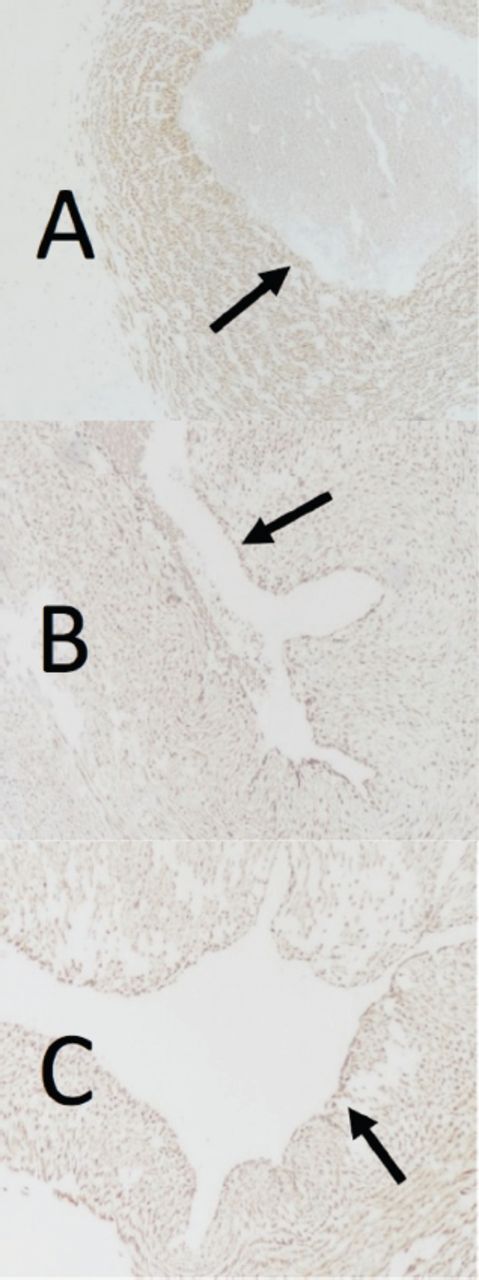
- The expression of fetuin-A in human umblical vein endothelial cells of gestational diabetes mellitus group. A) weak expression (+1), B) moderate expression (+2), C) strong expression (+3).

**Figure 2:**
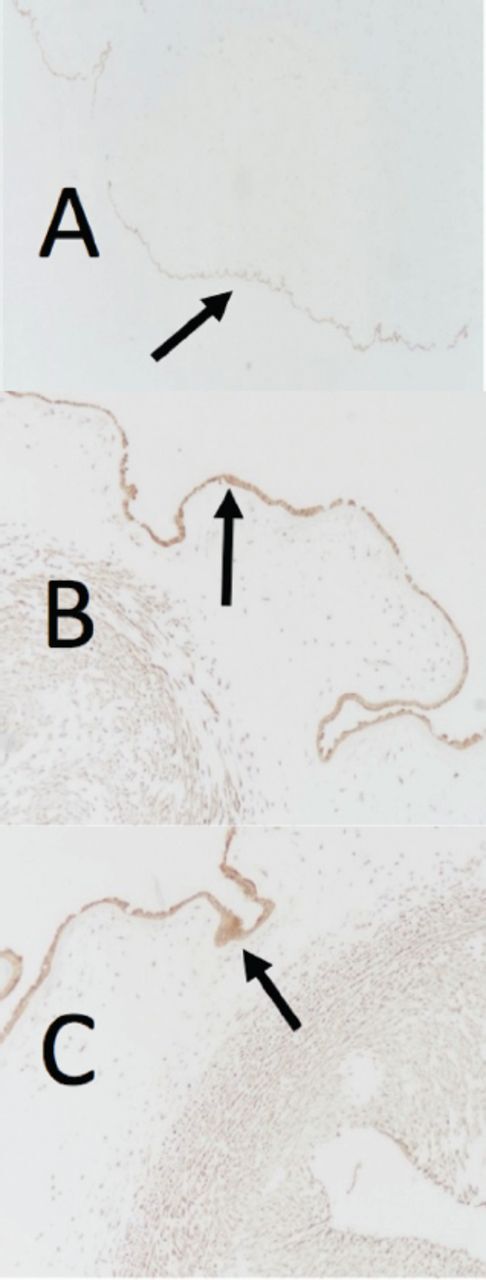
- The expression of fetuin-A in amnion cells of gestational diabetes mellitus group. A) weak expression (+1), B) moderate expression (+2), C) strong expression (+3).

Then all slides were stained with Fetuin-A and those were evaluated and scored simultaneously by 2 histopathologist who were aware of to the clinical diagnosis. The expression of fetuin-A in the GDM and control group was compared based on immunohistochemistry (IHC) staining in per randomly selected slides by the help of image capture system. The IHC staining intensity was evaluated by the density of dots which corresponds the immunostaining level of fetuin-A (fetuin score) both in HUVECs and ACs (Figure 1 & 2).

### Statistical analysis

The sample size was calculated by comparing preliminary data of fetuin-A expression in HUVECs for 10 patients for each GDM group and control group. The requisite sample size for those groups were established as 40 patients for each. The desired significance level was set at α=0.05 and power was set 0.8 (1-Beta) for a ratio of 1:1.

All statistical tests were carried out using MedCalc Statistical Software Programme, version 19.2 (Ostend, Belgium). The variables between GDM and control group were compared with Mann-Whitney-U and Chi-squared tests. The correlation of variables were calculated by Spearman’s rank correlation. The statistically significance determined as *p*<0.05.

## Results

Placental tissue and amnion membranes were retrieved from 82 women (GDM group=40 and control group=42) and there were no significant difference in regard of age, parity, gestational age, fetal macrosomia, neonatal hypoglycemia, fetal birth weight, and developing preeclampsia between the groups. Actually, the levels of fasting glucose levels were different between GDM (89.2±33.3 mg/dL) and control group (78±34.1 mg/dL) but the values did not reach the statistical significance. Clinical characteristics of the study groups were summarized in Table 1.

**Table 1:** - Clinical charateristics of study groups.

Patients characteristics	GDM (n=40)	Control (n=42)	*P*-value
Age (years), mean±SD	30.4±4.6	29.8±6.1	0.5972^*^
Gravidity, n (range of birth)	3 (1-4)	2 (1-5)	0.4076^**^
Parity, n (range of birth)	1 (0-4)	1 (0-3)	0.1593^**^
Fasting blood glucose (mg/dL), mean±SD	89.2±33.3	78±34.1	0.1413^**^
Pregnancy age (weeks)	38 weeks + 4 days	39 weeks + 2 days	0.1171^**^
Fetal macrosomia^†^	4 (10.0)	1 (2.4)	0.1520^††^
Preeclampsia^‡^	4 (10.0)	2 (4.8)	0.3655^††^
Neonatal hypoglycemia^§^	5 (12.5)	2 (4.8)	0.2128^††^
Fetal weight (g), mean±SD	3437±522	3205±493	0.2424^*^
Placental weight (g), mean±SD	541.2±283.1	456.3±318.7	0.2055^*^

Values are presented as number and precentage (%). ^†^Fetal macrosomia is defined as birth weight >4000 g.^25^^‡^According to The American College of Obstetricians and Gynecologists Preeclampsia Guideline 2020.^26^^§^According to American Academy of Pediatrics Neonatal Hypoglycemia Guideline.^27^^*^Independent samples t-test, ^**^Mann-Whitney test, ^††^Chi-squared test, GDM: gestational diabetes mellitus, SD: standard deviation

In the GDM group 7 patients needed insulin therapy and rest of the patients (n=33) reached the glycemic control with low-glycaemic index diets. Oral glucose tolerance test result of the GDM group were summarised in Table 2.

**Table 2:** - Oral glucose tolerance test results of the gestational diabetes mellitus group.

OGTT	n	Minimum (mg/dL)	Maximum (mg/dL)	Mean±SD (mg/dL)	Median (mg/dL)	25^th^ - 75^th^ percentile (mg/dL)
0 hour	40	76	111	86.125±8.3978	87	79-91
1 hour		172	199	188.375±7.4857	189	186-192
2 hours		154	177	163.800±8.3856	165	156-167
3 hours		131	155	140.275±8.7618	138	133-144

OGTT: oral glucose tolerance test, SD: standard deviation

The placental tissue of patients with GDM displayed significantly higher expression of fetuin-A which is not only in HUVECs (*p*<0.0001) but also in ACs (*p*=0.0001) when compared with the control group (Table 3).

**Table 3:** - Fetuin-A expression of human umblical vein endothelial cells and amnion cells in gestational diabetes mellitus and control group.

Variables	GDM (n=40)^^*^^				Control (n=42)				*P*-value
Fetuin scores	0	1+	2+	3+	0	1+	2+	3+	
Amnion cells	3	13	17	7	17	19	3	3	0.0001
HUVECs	4	7	13	16	13	24	3	2	<0.0001

* Chi-squared test, GDM: gestational diabetes mellitus, HUVECs: human umblical vein endothelial cells

Regarding the correlation between fetuin-A expression and clinicopathological variables, the expression of fetuin-A in HUVECs was correlated with fetal macrosomia (*p*=0.0117), neonatal hypoglycemia (*p*=0.0022), and placental weight (*p*=0.0309). On the other hand, there was no correlation between the expression of fetuin-A in ACs and clinicopathological variables (Table 4).

**Table 4:** - Spearman’s rank correlation analysis between Fetuin-A expression and clinicopathological variables.

Variables	HUVECs	ACs
Age	0.08814	0.03678
	p=0.5886	p=0.8218
Parity	0.01817	0.2016
	p=0.9114	p=0.2122
Fetal macrosomia^^*^^	0.3947	0.2448
	p=0.0117	p=0.1279
Preeclampsia^†^	-0.08664	-0.07598
	p=0.5950	p=0.6412
Neonatal hypoglycemia^‡^	0.4694	0.2202
	p=0.0022	p=0.1722
Fetal weight (g)	0.1779	0.2954
	p=0.2721	p=0.0643
Placental weight (g)	0.3226	0.1623
	p=0.0309	p=0.2674

* Fetal macrosomia is defined as birth weight >4000 g.^25^^†^According to The American College of Obstetricians and Gynecologists Preeclampsia Guideline 2020.^26^^‡^According to American Academy of Pediatrics Neonatal Hypoglycemia Guideline.^27^ HUVECs: human umblical vein endothelial cells, ACs: amnion cells, g: grams

## Discussion

Gestational diabetes mellitus is actually the most common medical complication of pregnancy, and prevalence of undiagnosed hyperglycaemia and even overt diabetes in young women is increasing due to obesity pandemic.^16^

Fetuin-A is a substantial component of metabolic syndrome, insulin resistance, and is related with type 2 diabetes.^17,18^ Fetuin-A exerts its direct effects on glucose metabolism by binding the outer portion of insulin receptor and prevents the autophosphorylation of tyrosine kinase and also blocks insulin-stimulated GLUT4 translocation.^12,17^ The strong correlation between fetuin-A and insulin resistance was exposed with a meta-analysis in which it was revealed that fetuin-A levels were remarkably high in type 2 diabetic patients.^17^ On the other hand, in another meta-analysis; it was speculated that fetuin-A levels in patients with GDM were not significantly different from those in the control group during the first or early second trimester of pregnancy.^18^ However, the role of fetuin-A in the placenta is obscure.

Fetuin-A signifies its stimulatory effects on inflammatory responses in human umblical veins, leading to the development of atherosclerosis and may inhibit placental cell growth and ciliogenesis in GDM.^12,19^ Moreover, the mothers of macrosomic infants had lower fetuin-A levels than the mothers of normal weight babies.^13^ Conversely, in a nested case-control study; it was disclosed that fetal cord fetuin-A levels were higher both small for gestational age (birth weight <10^th^ percentile) and large for gestational age (LGA, birth weight >90^th^ percentile) infants when compared with optimal for gestational age (birth weight between 25-75^th^ percentiles) infants. However only LGA infants had elevated odds risk for fetuin-A after adjusted for fetal and maternal parameters irrespective of fetal growth factors.^20^

It has been revealed that placental fetuin-A concentration increases in patients with GDM and the mRNA level of fetuin-A increased in the placental tissue of patients with GDM and it is speculated that fetuin-A was also synthesized locally.^12^ It was uncovered in our study that the local expression of fetuin-A is significantly higher in ACs and HUVECs of patients with GDM. Thereby, both circulating and local placental fetuin-A affect placental development in patients with GDM in a coordinated manner.

A clinical research elucidated that there is a relationship between human fetuin-A and pregnancy outcomes in GDM, in favor of worser consequences. This is a promising data for the detection of abnormalities after birth in babies born from GDM mothers and also after birth of GDM patients. It is well-known that GDM mothers have a higher risk of diabetes during their life and fetuin-A may affect this metabolic process from various pathways.^21^

In some in vitro experiments with extravillous trophoblast cells, it has been uncovered that fetuin-A may decrease trophoblast viability and invasion caused by the inhibition of receptor tyrosine kinase activity.^22^ It has been unveiled that elevated serum levels of fetuin-A might be associated with preeclampsia and fetal growth restriction.^22-24^

In our study, there is a correlation between fetal macrosomia, neonatal hypoglycemia, placental weight, and fetuin-A expression of HUVECs in GDM patients. Additionally, an excursion on fetuin scores can be conferred in both ACs and HUVECs of GDM patients. The expression of fetuin might be linked in the development of GDM.

### Study limitation

The method we used to show expression of fetuin-A in placental tissue which is replaced by new techniques such as real time PCR.

In conclusion, the expression of fetuin-A may have some predictive value for the occurrence of adverse outcomes in women with GDM. There is only limited data in literature which explores the tissue expression of fetuin-A in HUVECs and ACs of GDM patients. Our study may provoke the design of other studies on GDM and the tissue expression of fetuin-A.
